# Communities of microbial eukaryotes in the mammalian gut within the context of environmental eukaryotic diversity

**DOI:** 10.3389/fmicb.2014.00298

**Published:** 2014-06-19

**Authors:** Laura Wegener Parfrey, William A. Walters, Christian L. Lauber, Jose C. Clemente, Donna Berg-Lyons, Clotilde Teiling, Chinnappa Kodira, Mohammed Mohiuddin, Julie Brunelle, Mark Driscoll, Noah Fierer, Jack A. Gilbert, Rob Knight

**Affiliations:** ^1^Biofrontiers Institute, University of ColoradoBoulder, CO, USA; ^2^Department of Molecular, Cellular, and Developmental Biology, University of ColoradoBoulder, CO, USA; ^3^Cooperative Institute for Research in Environmental Sciences, University of ColoradoBoulder, CO, USA; ^4^454 Life Sciences, Roche CompanyBranford, CT, USA; ^5^Department of Ecology and Evolutionary Biology, University of ColoradoBoulder, CO, USA; ^6^Department of Ecology and Evolution, University of ChicagoChicago, IL, USA; ^7^Institute of Genomic and Systems Biology, Argonne National LaboratoryArgonne, IL, USA; ^8^Howard Hughes Medical Institute, University of ColoradoBoulder, CO, USA

**Keywords:** protist, microbial ecology, microbial diversity, salinity, host-associated eukaryotes, parasites, intestinal protozoa, human microbiome

## Abstract

Eukaryotic microbes (protists) residing in the vertebrate gut influence host health and disease, but their diversity and distribution in healthy hosts is poorly understood. Protists found in the gut are typically considered parasites, but many are commensal and some are beneficial. Further, the hygiene hypothesis predicts that association with our co-evolved microbial symbionts may be important to overall health. It is therefore imperative that we understand the normal diversity of our eukaryotic gut microbiota to test for such effects and avoid eliminating commensal organisms. We assembled a dataset of healthy individuals from two populations, one with traditional, agrarian lifestyles and a second with modern, westernized lifestyles, and characterized the human eukaryotic microbiota via high-throughput sequencing. To place the human gut microbiota within a broader context our dataset also includes gut samples from diverse mammals and samples from other aquatic and terrestrial environments. We curated the SILVA ribosomal database to reflect current knowledge of eukaryotic taxonomy and employ it as a phylogenetic framework to compare eukaryotic diversity across environment. We show that adults from the non-western population harbor a diverse community of protists, and diversity in the human gut is comparable to that in other mammals. However, the eukaryotic microbiota of the western population appears depauperate. The distribution of symbionts found in mammals reflects both host phylogeny and diet. Eukaryotic microbiota in the gut are less diverse and more patchily distributed than bacteria. More broadly, we show that eukaryotic communities in the gut are less diverse than in aquatic and terrestrial habitats, and few taxa are shared across habitat types, and diversity patterns of eukaryotes are correlated with those observed for bacteria. These results outline the distribution and diversity of microbial eukaryotic communities in the mammalian gut and across environments.

## Introduction

A rich understanding of the distribution of microbial diversity across environments has emerged from high-throughput sequencing studies in the past decade. These studies have described many spatial and temporal patterns of variability within environments and have defined the major divisions in microbial community composition (Nemergut et al., 2013). Salinity represents the primary division among environmental samples for bacterial and archaeal communities (Lozupone and Knight, 2007; Auguet et al., 2010; Wang et al., 2011), while the vertebrate gut has the most distinct bacterial communities (Ley et al., 2008b). Studies characterizing microbial diversity deeply across hundreds to thousands of samples are now common for bacteria (e.g., the Human Microbiome Project, the Earth Microbiome Project, MetaHIT), but are just beginning for microbial eukaryotes (Tara Oceans, ICOMM, BioMarks). As a result, progress characterizing the distribution of protist diversity lags behind our knowledge of bacteria, but morphological surveys (Larsen and Patterson, 1990; Patterson, 1996; Foissner, 2006; Weisse, 2008) combined with recent molecular data (Amaral-Zettler et al., 2009; Caron, 2009; Baldwin et al., 2013; Bates et al., 2013) provide a foundation of knowledge on the biogeography of protists across environments.

Our understanding of the diversity and function of host-associated microbial communities has grown exponentially in recent years, fueled by high-throughput sequencing and motivated by the realization that microbes have a profound influence on their host (McFall-Ngai et al., 2013; Sommer and Backhed, 2013). There are many commonalities in the bacterial taxa that comprise the microbiota across mammals, with the phyla Bacteroidetes and Firmicutes being predominant components (Ley et al., 2008b; Muegge et al., 2011). Overall, the mammalian gut harbors lower bacterial diversity and fewer phyla-level taxa than other environments (Ley et al., 2006). Across mammals, microbiota composition varies according to host phylogeny and diet (Ley et al., 2008b; Russell et al., 2014), and the composition of the human microbiota resembles that of our primate relatives (Ley et al., 2008b). Within humans gut microbiota is influenced by diet, health status, and age (Fierer et al., 2012; Lozupone et al., 2012). In addition, adoption of a western lifestyle, characterized by diets rich in processed food, antibiotic usage, and hygienic habits, has a particularly strong influence on the microbiota (De Filippo et al., 2010; Yatsunenko et al., 2012; Ursell et al., 2013). Diversity of the human bacterial microbiota has clearly declined in Western populations compared to populations with traditional agrarian lifestyles (De Filippo et al., 2010; Cho and Blaser, 2012; Lozupone et al., 2012; Yatsunenko et al., 2012).

Progress characterizing the eukaryotic component of the mammalian microbiome lags behind bacteria because high-throughput sequencing based investigations into the diversity of the mammalian microbiota have focused almost exclusively on bacteria (Parfrey et al., 2011; Andersen et al., 2013). The mammalian intestinal tract is home to many eukaryotes, including animals (e.g., helminths) and protists (e.g., amoebae and flagellates), and these taxa have been investigated for decades from a parasitological point of view with microscopy and targeted molecular approaches (Bogitsh et al., 2005). Studies of the eukaryotic component of the mammalian microbiota from a community perspective are beginning to come online, though many questions remain to be investigated (Andersen et al., 2013). Although sample sizes are generally small to date, these studies have shown that anaerobic fungi are dominant in mice (Scupham et al., 2006). Western human fecal communities include *Blastocystis* (Scanlan and Marchesi, 2008) and fungi (Dollive et al., 2012), while a survey of a single African individual revealed higher microbial eukaryote diversity (Hamad et al., 2012). The diversity of the eukaryotic microbiota in the human gut has not yet been systematically investigated from a community perspective in non-western populations. These populations provide an important perspective for understanding the eukaryotic microbiota that humans have co-evolved with over millions of years.

Eukaryotic microbes in the gut are generally considered parasites, and have long been recognized to contribute to host morbidity and mortality (Bogitsh et al., 2005). However, many are commensal (Bogitsh et al., 2005), or play beneficial roles as probiotics (McFarland and Bernasconi, 1993) or cellulose degraders (Kittelmann and Janssen, 2011). Further, increasing evidence suggests that eliminating the diverse microbial community that co-evolved with mammals over millions of years is detrimental to host health (Cho and Blaser, 2012; Lozupone et al., 2012), in support of the Old Friends Hypothesis (or hygiene hypothesis) (Rook, 2012). Eukaryotic microbes were part of our ancestral gut community and intestinal helminths were nearly universal (Goncalves et al., 2003). In humans, the transition to modern lifestyles is associated with dramatically lower diversity and prevalence of intestinal helminths, and with a rise in the prevalence of autoimmune disease (Rook, 2012). Yet, we know little about their role in healthy people. Recent analyses of common protists in the gut suggests that they may be part of the healthy microbiota in humans (Petersen et al., 2013).

Here, we use high-throughput sequencing to characterize eukaryotic communities found in the vertebrate gut from a diverse collection of mammalian fecal samples, including humans from the US and from remote communities in Malawi. To provide a broader context for understanding of the diversity of microbial eukaryotes in the gut, we also characterized a collection of samples from a wide range of other environments, including human skin, marine water, freshwater, soil, and air. The bacterial communities in these samples were also characterized to enable comparison of eukaryotic and bacterial biodiversity. In order to gain deeper insight into the distribution of eukaryotic diversity, we curated the SILVA reference database (Pruesse et al., 2007) so that both the taxonomy assigned to reference sequences and the phylogenetic tree constructed from these reference sequences reflects current knowledge. Eukaryotic environmental sequences are placed within this explicit phylogenetic context and assess the distribution of eukaryotic clades across environments.

## Methods

### Sample set

We selected 185 samples that span a wide range of environments in order to assess broad patterns in eukaryotic communities (Table S1). The dataset analyzed here was chosen to include individuals from geographically diverse populations with contrasting lifestyles to enable testing the hypothesis that the transition to modern, highly hygienic lifestyles are correlated with low levels of diversity of eukaryotic microbes. We included samples from 23 individuals that reside in agrarian communities in Malawi that follow traditional lifestyles and 16 samples from 13 individuals residing in the US (Boulder, CO and Philadelphia, PA) and follow modern lifestyles (Table 1). Three individuals from Boulder were sampled at two time points 2 months apart (Costello et al., 2009). The US populations live in urban or suburban areas, consumed typical western diets, and did not report any health problems at the time of sampling (Costello et al., 2009; Yatsunenko et al., 2012). Individuals from populations in Malawi ate diets rich in maize, legumes, and other plants (Table S1 from Yatsunenko et al., 2012) and were healthy and well-nourished at the time of sampling (Yatsunenko et al., 2012; Smith et al., 2013). These samples have been described in detail previously and bacterial diversity was previously reported (Costello et al., 2009; Yatsunenko et al., 2012; Smith et al., 2013). In addition, we included 22 samples from other mammals, also previously described and characterized for bacteria (Ley et al., 2008a; Muegge et al., 2011), to gain insight into the diversity of eukaryotic human microbiota relative to other mammals. Collection of the human fecal samples for these previously published studies was done according to protocols approved by Human Research Committees at the institutions involved which allow samples to be used for further research. De-identified DNA was sent to the University of Colorado for amplification. Collection of skin and oral samples was approved by the University of Colorado Human Research Committee (protocol 0109.23), which allows the samples to be used for further research. Finally, we included samples from wide variety of environments, many of which have been previously characterized for bacterial or fungal communities (Table S1). These include air sampled over terrestrial environments (Bowers et al., 2011a, 2012), soil (Lauber et al., 2009; Ramirez et al., 2010; Eilers et al., 2012), freshwater (Shade et al., 2012), marine water, lichens (Bates et al., 2011), leaf litter (McGuire et al., 2012), and human oral and skin samples (Costello et al., 2009; Verhulst et al., 2011). The sequence data and MiMARKs (Yilmaz et al., 2011) compliant metadata is available for this study at the QIIME database (http://www.microbio.me/qiime/: study #1519 for eukaryotes and #1517 for bacteria) and at EBI (accession numbers ERP006039 and ERP005135).

**Table 1:** **Human fecal samples**.

**Sample name**	**Village^a^**	**Age (years)**	**Original study^b^**	**Total seqs**	**Filtered seqs^c^**	***Blastocystis***	***Entamoeba***
h101M	M: Mbiza	24.4	2	1041	982	ST3	*coli, hartmanii*
k57B.6Post	M: M: Mbiza	2.0	1	649	638	ST3	*coli*
h101A.4	M: Mbiza	2.3	2	719	546	ST3	
h101B.4	M: Mbiza	2.3	2	901	521	ST3	
k84M	M: Mayaka	30.6	1	821	493	ST1, ST3	*coli, dispar, hartmanii*
h186M	M: Mayaka	31.6	2	400	367	ST1, ST3	*coli, hartmanii*
k26M.1	M: Mitondo	29.5	1	929	361	ST3	*coli, dispar, histolytica, hartmanii*
h186A.1	M: Mayaka	2.0	2	1024	305		*coli*
h146B.2	M: Mayaka	1.7	2	319	261	ST2, ST3	*hartmanii*
h146M	M: Mayaka	33.5	2	246	233	ST1, ST3	*hartmanii*
m55M	M: Mbiza	adult	1	277	229	ST1, ST2	*coli, hartmanii*
k57M	M: Mbiza	30.8	1	233	212	ST1, ST2, ST3	*coli*
k80M	M: Mayaka	27.2	1	256	168	ST1	*coli*
m55S	M: Mbiza	child	1	263	165	ST2	*coli, hartmanii*
h47M	M: Chamba	adult	2	526	144	ST2	*coli*
k80A.7	M: Mayaka	1.9	1	285	118		*dispar, histolytica*
k84A.1	M: Mayaka	0.9	1	774	45	ST1, ST2, ST3	
h186B.1	M: Mayaka	2.0	2	900	44		*hartmanii*
h146A.2	M: Mayaka	1.7	2	935	10	ST3	*hartmanii*
h18A.3	M: Chamba	1.1	2	1028	8		*coli*
h47A.1	M: Chamba	0.6	2	1032	6		
h47B.1	M: Chamba	0.6	2	400	3		
h18B.5	M: Chamba	1.6	2	193	1		
USBldChld5	U: Boulder	3	2	485	123		*coli*
USchp60Mom	U: Philadelphia	33	2	1006	47		
USchp18Child	U: Philadelphia	3	2	977	35		
USBldChld8	U: Boulder	1.6	2	671	29		*coli*
M22Fcsw	U: Boulder	adult	3	935	14		
USBldChld4	U: Boulder	6	2	1159	7		
M21Fcsw	U: Boulder	adult	3	825	5		
USBldChld10	U: Boulder	1.3	2	913	1		
USBldChld2	U: Boulder	4.5	2	492	0		
USchp33ChildA	U: Philadelphia	5	2	378	0		
USchp33Mom	U: Philadelphia	45	2	781	0		
F11Fcsw	U: Boulder	adult	3	139	0		
M11Fcsw	U: Boulder	adult	3	502	0		
M23Fcsw	U: Boulder	adult	3	156	0		
M24Fcsw	U: Boulder	adult	3	221	0		
M31Fcsw	U: Boulder	adult	3	269	0		

a Country where village is located: M, Malawi and U, USA.

b Original study: 1 = Smith et al., 2013; 2 = Yatsunenko et al., 2012; 3 = Costello et al., 2009.

c Filtered sequences have the following removed: Bacteria, Archaea, non-18S rDNA, mammalian DNA, plants.

### Microbial community characterization

Sequences were PCR amplified with primers 515f and 1119r (Bates et al., 2012). The forward primer 515f (5' GTGCCAGCMGCCGCGGTAA 3') is 3-domain universal and 1119r (5' GGTGCCCTTCCGTCA 3') is targeted toward eukaryotes. Primer specificity to eukaryotes and predicted amplification efficiency of eukaryotic lineages was assessed with the taxa coverage module in PrimerProspector (Walters et al., 2011). This program assesses the complementarity between the primer sequence and a reference database, in this case SILVA 111, and assigns a score based on the number of mismatches or gaps between the primer sequence and the reference, and mismatches as the 3' end of the primer are more heavily penalized (http://pprospector.sourceforge.net/tutorial.html). Taxa coverage was assessed at three thresholds corresponding to three levels of specificity (Table S2). A threshold of 0.5 is predicted to generate efficient amplification and allows up to one mismatch at the 5' end of the primer. The threshold of 1 allows one mismatch at the 3' end of the primer or two mismatches in other primer regions, and threshold 2 allows 2–5 mismatches at the 3' or 5' ends of the primer respectively and amplification is expected to be poor or non-existent. This primer pair has high predicted specificity to eukaryotes, matching 86–90% of eukaryotic sequences but less than 0.5% of bacterial and archaeal sequences at a threshold of 0.5 and 1, respectively (Table S2). Many of the taxa expected to be in the mammalian gut based on parasitological studies are predicted to amplify well, including *Dientamoeba, Entamoeba, Blastocystis, Balantidium*, parabasalids, and nematodes (Table S2). However, there are two mismatches between the *Giardia* 18S sequence and the reverse primer suggesting a low efficiency (Table S2).

DNA was extracted with the MoBio PowerSoil kit following EMP standard protocols. PCR amplification was done in triplicate with an annealing temperature of 50C for 40 cycles. These permissive conditions were used to amplify the broadest range of eukaryotic taxa. Quantitation and pooling were done according to EMP standard protocols. The final pool was sent to Roche Core Facility. The libraries were amplified, sequenced and processed at the Roche Core Facility. Amplification was done according to the emPCR Amplification Method Manual—Lib-A LV GS FLX Titanium Series with the following edits for long amplicons. Using the Titanium Lib-A emPCR kit, the emulsions were made with A beads and A amp primers only and the following reagents: 1050 μL MBGW, 1500 μL emPCR additive, 860 μL 5× amplification mix, 300 μL Primer (A), 200 μL Enzyme mix, and 5 μL PPiase. The cycling conditions were 4 min at 94C followed by 50 cycles of 30 s at 94C and 10 min at 60C, ending with a hold at 10C. The library was then run as a standard XL+ run. This FLX+ run was sequenced with the standard flow order (400 cycles of TACG nucleotide flows), following the instructions in the Sequencing Method Manual—GS FLX+ Series—XL+ kit, as can be found on the www.my454.com website.

### Data processing and quality filtering

Data processing was done at the Roche Core Facility according to the GS FLX System Software Manual modified to optimize performance for metagenomic amplicon sequences. In order to generate high quality data for amplicons metagenomic applications, the default pipeline was tuned to meet the data quality requirements of the QIIME pipeline. The data was processed using 26amp_sl1000 pipeline which has the following tuning steps modified: (1) vfScanLimit was increased from the default of 700 to 1000, (2) the valley filter setting vfTrimBackScaleFactor was increased from the default value by a factor of 0.5, and (3) the quality filter setting QscoreTrimFactor was modified from the default value to a more stringent value. The Amplicon pipeline template was used to generate the modified pipeline XML file with the rCAFIE algorithm turned on.

Usearch version 6.1 was used to screen sequence for chimeras (Edgar, 2010). Sequences were additionally filtered for quality using split_libraries within QIIME version 1.5.0 (Caporaso et al., 2010b). Quality filtering excluded sequences with an average quality score of 25 or lower, reads longer than 1200 bp or shorter than 200 bp and reads with more than 5 ambiguous bases. We found that sequence quality dropped off significantly toward the end of the read, so we employed a strategy truncating sequences when quality scores that fell below 25 in a sliding window of 50 bp. These truncated reads were retained as long as they passed other quality filters and these averaged 444 bp in length.

In order to quantify concordance in the diversity patterns of bacterial and eukaryotic communities we sequenced the bacterial communities as well as the eukaryotic communities. Bacteria were sequenced with the 515f/806r primers (Walters et al., 2011) on the Illumina GAIIx platform at Washington University. Bacterial data was processed using standard protocols within the QIIME database (www.microbio.me/qiime). Archaea are also amplified with this primer set, but were excluded from the analysis in order to focus on the eukaryote to bacteria comparison and because there were too few Archaea OTUs for meaningful comparison. Low abundance OTUs, those containing less than 0.05% of the total reads in the dataset, were filtered out as recommended for Illumina sequence data (Bokulich et al., 2013). The samples were filtered to only include those 113 samples that had at least 150 sequences per samples in the eukaryotic data, and of these, samples with fewer than 3000 sequences were excluded from the analysis. The full dataset was used for taxon-based analyses and all samples were rarefied to 3000 sequences per sample for diversity analyses.

### OTU picking and taxonomy assignment

Eukaryotic sequence reads from the 454 FLX+ run were clustered into OTUs with a 97% similarity threshold, which was chosen to minimize the impact of sequencing error in inflating OTU numbers (Stoeck et al., 2010; Bates et al., 2013). Reads were clustered into OTUs according to the open reference protocol (http://qiime.org/tutorials/open_reference_illumina_processing.html) using UCLUST (Edgar, 2010) within QIIME. This involves first clustering reads against the curated SILVA 108 eukaryotic database clustered at 97%, and these OTUs inherited the reference taxonomy. Sequences that failed to assign to the reference dataset were then clustered at 97% *de novo* with UCLUST. Taxonomy was assigned to these *de novo* sequences in one of two ways in order to maximize the taxonomic information and reliability. First, taxonomy was assigned using BLAST against the SILVA 108 97% reference database with an *e*-value cutoff of e-100. In cases where the *e*-value was less than e-100 taxonomy was assigned using the RDP classifier trained with the SILVA 108 97% reference set at genus level. Taxonomy assignments were also confirmed in using the PR2 reference database (Guillou et al., 2013). The resulting OTUs were filtered to exclude bacteria, archaea, vertebrates (thus removing host DNA), and plants (to exclude dietary sources) as well as non-SSU rDNA sequences. Finally, singleton sequences were excluded from the analysis to reduce the likelihood of including PCR and sequencing artifacts. After filtering, we excluded samples from further analysis that had fewer than 150 eukaryotic sequences/sample. This left 3883 OTUs from 113 samples (out of 185 total samples), corresponding to 84,576 sequences. Downstream diversity analyses used data rarefied to 150 sequences per sample, and taxonomy plots used the full dataset. In order to take full advantage of this dataset we assessed the taxonomic composition of human gut samples falling below the 150 sequences per sample threshold. In this case, a taxon (OTU) was considered present if the OTU was represented by least 5 sequences in the sample in question.

Although 150 sequences per sample is a low number by high-throughput sequencing standards, this sequencing depth adequately captures the diversity present (Figure S1). Direct comparison of numbers of bacterial and eukaryotic taxa is not possible because two different sequencing platforms were used here and the number of sequences per sample is much lower for eukaryotes. However, we can compare the relative differences in alpha diversity between sample types for eukaryotes and bacteria respectively, and sequencing depth for both domains adequately sample diversity. Rarefaction curves of Faith's Phylogenetic Diversity metric level off by 150 sequences per sample, particularly for host-associated samples (Figure S1). Similarly, we have adequate sampling of bacterial diversity and rarefaction curves are leveling off by 3000 sequences per sample for host-associated samples (Figure S1).

A phylogenetic tree reflecting the current understanding of eukaryotic relationships was constructed using the curated SILVA alignment as a template and the SILVA 108 tree as a constraint on the backbone relationships (see SILVA curation below). The representative set of sequences from this study was first aligned to the SILVA 108 97% representative set with PyNAST (Caporaso et al., 2010a). Representative sequences for each of the 3883 OTUs that aligned to the SILVA reference alignment were used to build a phylogenetic tree for diversity analysis and to assess patterns of phylogenetic groups by environment. The resulting alignment was dynamically filtered to remove the 10% most entropic positions and positions with greater than 95% gaps. This alignment was then used to build a phylogenetic tree with the topology constrained to the SILVA 108 97% tree (see below) in RAxML (Stamatakis, 2006). This tree was used for visualization in TopiaryExplorer (Pirrung et al., 2011), which allows branches to be colored according to sample metadata or taxonomy. The *p*-test from Martin (2002) and UniFrac test (Lozupone and Knight, 2005) were performed on the tree to assess whether the distribution of sequences from particular environments across the tree were significantly different than random, implemented in the beta_significance script within QIIME. In order to visually compare the diversity in the vertebrate gut to other environments, we filtered the tree to include equal sample numbers and equal (rarefied) sequences per sample. This was done by first filtering the OTU table to include the 32 fecal samples with more than 150 sequences per sample and a subsampled set of 32 environmental samples spanning the range of environments, and then rarefied to 150 sequences per sample for both eukaryotic 18S and bacterial 16S. This normalized OTU table was used to filter tips from the 16S and 18S trees.

Diversity analyses were carried out in QIIME using data rarefied to 150 sequences per sample for eukaryotes and 3000 sequences per sample for bacteria. The differences in rarefaction level are a result of the different sequencing platforms used for these datasets. Phylogenetically informed analyses of alpha and beta diversity [phylogenetic distance and unweighted UniFrac (Lozupone and Knight, 2005), respectively] utilized the tree described above. Non-phylogenetic beta diversity metrics performed poorly because very few OTUs were found across multiple sample types (Table 2). Unweighted UniFrac distance matrices were used in Analysis of variance tests (ANOSIM) to assess statistical differences across environments within QIIME. To assess the impact of unbalanced numbers of samples across habitat types, we randomly subsampled the dataset to include equal numbers of samples from each environment and then recalculated diversity metrics and performed ANOSIM tests. This procedure was repeated 1000 times. We visualized the differences in beta-diversity across sample types with non-metric multidimensional scaling (NMDS) plots, which were constructed in the software Primer E (Clarke and Gorley, 2006).

**Table 2:** **Proportion of shared eukaryotic OTUs**.^*^

**Environment**	**Fecal**	**Skin**	**Terrestrial**	**Freshwater**	**Marine**
Fecal	190	1	3	1	0
Skin	1	68	34	6	1
Terrestrial	3	34	1796	80	2
Freshwater	1	6	80	354	4
Marine	0	1	2	4	482
Total OTUs	190	68	1796	354	482
% Unique	97%	38%	93%	74%	99%

* Calculations were done based on the full dataset, and exclude fungi. Fungi have low taxonomic resolution for 18S rRNA (Schoch et al., 2012), thus shared fungal 97% OTUs may be quite divergent.

We took advantage of the long sequence reads from the 454 FLX+ to further investigate the phylogenetic position of *Entamoeba* and *Blastocystis*, the two most common taxa detected in the gut. We aligned *Entamoeba* and *Blastocystis* representative sequences to the reference taxa from the PR2 database, and then constructed maximum likelihood phylogenies with RAxML. These trees were constrained to the reference phylogeny for these clades, which was derived from the literature (Stensvold et al., 2011; Alfellani et al., 2013). The placement of *Entamoeba* and *Blastocystis* sequences was used to confirm the taxonomic identities of these OTUs (Table 1).

### Curation of the SILVA eukaryotic database

The SILVA 108 ribosomal database (Pruesse et al., 2007) was downloaded from SILVA (http://www.arb-SILVA.de/). Sequences were initially filtered to remove unclassified environmental sequences. The remaining ~55,000 sequences were dereplicated by clustering at 97% with UCLUST, resulting in ~11,000 sequences. A representative set was then chosen for these OTUs based on the longest sequence. The filtered out environmental sequences were then clustered against the representative set of 97% OTUs using UCLUST ref within QIIME. Those sequences that did not match the reference dataset were then clustered at 97% *de novo* and the longest representative sequence chosen for each cluster. This resulted in a final SILVA eukaryotic 97% representative set with 14,236 sequences.

The 97% reference dataset was aligned with PyNAST (Caporaso et al., 2010a) in QIIME with a threshold of 70% similarity and a template alignment from Katz et al. (2011) [TreeBase study 11336, matrix M8584; (Katz et al., 2011)]. The resulting alignment was dynamically filtered to remove the 20% most entropic positions and positions with more than 90% gaps. A phylogenetic tree was constructed with RAxML version 7.3.0 (Stamatakis et al., 2008), using the tree topology from the multigene study of Parfrey et al. (2010) with updates based on subsequent papers (e.g., Adl et al., 2012) as a constraint.

The database taxonomy was curated to reflect current views of eukaryotic taxonomy and maximize the taxonomic information available for environmental sequences. Major clade information was added based on Parfrey et al. (2010) and Adl et al. (2012). To maximize the informativeness of the SILVA data set, high-level taxonomy was assigned to uncultured environmental sequences by placing these uncultured reads into the tree of SILVA representative sequences with the RAxML EPA algorithm (Berger et al., 2011) and assessing their position in a phylogenetic tree. Sequences that were nested within clades were assigned taxonomy based on that clade at a high level (e.g., Ciliate or Fungi). Sequences that were mislabeled (i.e., sequence labeled as fungi that fell within the plants) were identified in the tree, confirmed by BLAST and then removed from the representative set. The curated SILVA 108 database is available at http://qiime.org/home_static/dataFiles.html.

## Results and discussion

### Eukaryotic diversity in the human gut

**Figure 1:**
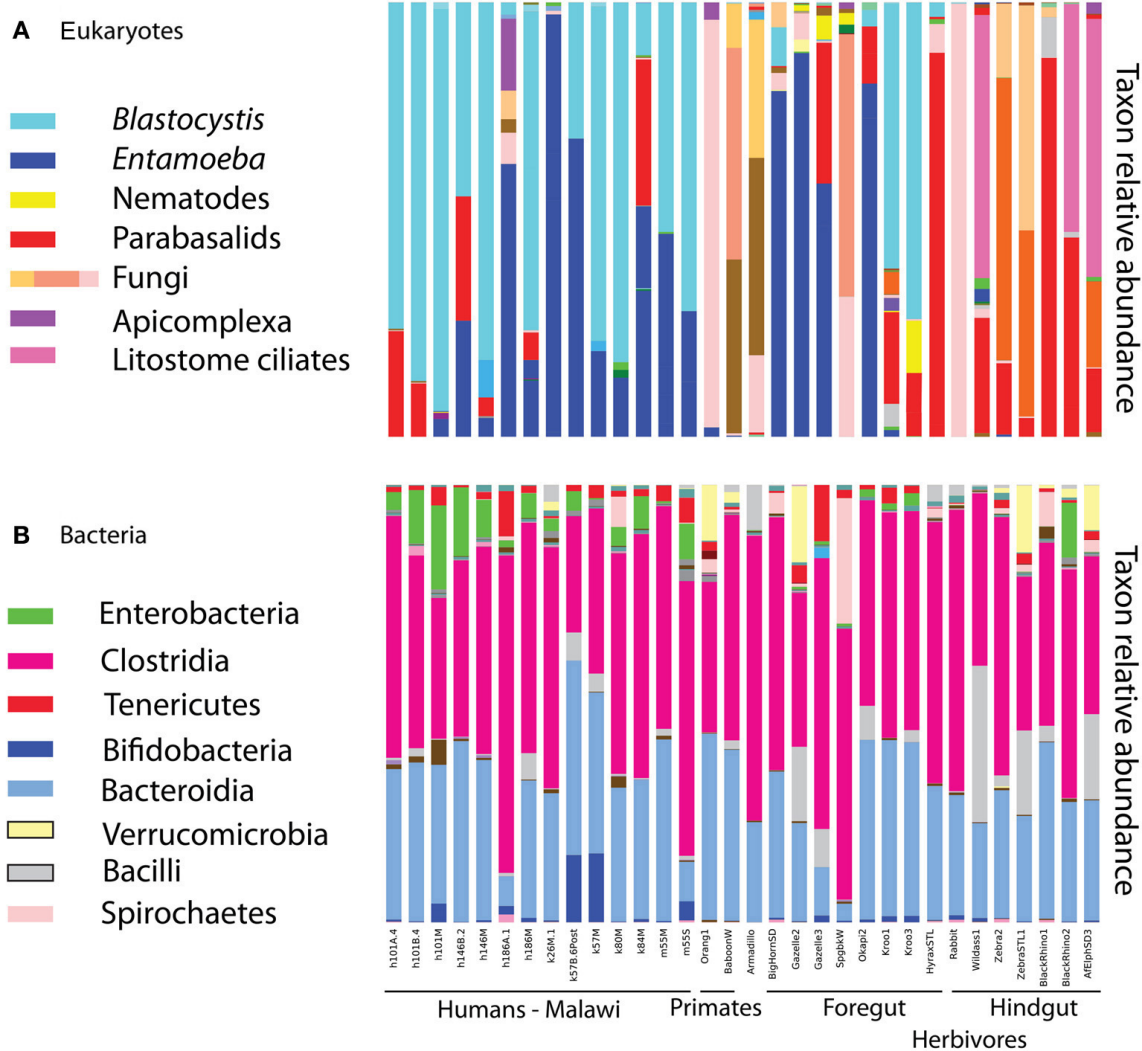
**Relative taxon abundance of mammalian (including human) fecal samples demonstrates heterogeneity in the presence of eukaryotic lineages across mammals, while the same bacterial lineages are consistently dominant. (A)** Eukaryotes, **(B)** bacteria. Each bar represents an individual fecal sample from humans and other mammals, and only samples with at least 150 sequences in the 18S are represented.

Eukaryotic microbes are common components of the human gut microbiota in healthy individuals. *Blastocystis, Entamoeba*, trichomonads, and yeast were frequently detected in human gut samples (Figure 1). Closer inspection of the taxa reveals that most are likely commensal rather than pathogens. For example, *Entamoeba* was detected in both populations. While the genus *Entamoeba* includes *E. histolytica*, the causative agent of the deadly amoebic dysentery (Bogitsh et al., 2005), the vast majority of *Entamoeba* sequences detected here fall within the commensal species *Entamoeba coli, E. dispar*, and *E. hartmanni* (Table 1). *Entamoeba histolytica* was detected in low abundance in two individuals that also harbored *E. dispar*.

*Blastocystis* was abundant in many samples (Figure 1), and represented by subtypes ST1, ST2, and ST3 (Table 1). Historically, *Blastocystis* has been considered a pathogen and it is associated with Irritable Bowel Syndrome (Yakoob et al., 2010; Poirier et al., 2012). However, the clinical importance of *Blastocystis*, its pathogenicity, and variation in pathogenicity among subtypes, is widely debated (Tan et al., 2010; Coyle et al., 2012; Scanlan and Stensvold, 2013). Some evidence suggests that *Blastocystis* is a normal component of the microbiota in many individuals—perhaps even a beneficial component—as it has been detected at high prevalence in healthy people (Scanlan and Marchesi, 2008; Petersen et al., 2013; Andersen et al., submitted), its presence is negatively correlated with intestinal disease (Petersen et al., 2013), but see Cekin et al. (2012). High prevalence of *Blastocystis* has been reported in other epidemiological studies of African countries, up to 100% reported in a Senegalese cohort, half of which had no gastrointestinal symptoms (El Safadi et al., 2014). Many other taxa that populate parasitology textbooks were also detected at lower levels, including *Chilomastix*, nematodes, and other parabasalids. We do not detect common gut symbionts such as *Dientamoeba* (Parabasalia), *Cryptosporidium* (Apicomplexa), or *Giardia* (Diplomonadida). The primers used here are a poor match for *Giardia* (Table S2) and may have failed to amplify *Giardia* DNA. The primers are predicted to work well with *Cryptosporidium*, but our DNA extraction method (bead beating rather than freeze thaw cycles) may have been insufficient to break open the robust spores of *Cryptosporidium* (and similar problems may further hinder our ability to detect *Giardia*). *Dientamoeba* is also predicted to amplify with our primers (Table S2). While prevalence is generally quite high in Europe, *Dientamoeba* prevalence is variable worldwide and generally low (less than 5%) in Africa (Barratt et al., 2011). However, specific diagnostic assays would be necessary to rule out presence of these taxa with any confidence.

We assessed eukaryotic diversity across two geographically distant populations whose inhabitants follow either traditional, agrarian lifestyles (Malawi) or modern, urban lifestyles (US). However, our ability to compare eukaryotic diversity across populations is hampered by low counts of eukaryotic sequences in US individuals and young children. Taxa presence above was calculated based on OTUs represented by at least five sequences in a given sample. In order to compare diversity across populations and across sample types more broadly, we filtered out samples with fewer than 150 eukaryotic sequences. While all but three human fecal samples had greater than 150 sequences per sample in total, 27 samples fell below this threshold after removing sequences from bacteria, host, and dietary plants. These non-target taxa account for 94–100% of the sequences from all but one US samples and most children age two and younger (Table 1). One samples from a three-year-old US child had a large portion of sequences derived from *Entamoeba coli*. The primer set used here targets eukaryotic 18S has a low affinity for vertebrate 18S sequences, and successfully amplified the eukaryotic community in most samples, including environmental samples and mammalian feces (Table S1). We suspect that the high proportion of non-target sequences amplified in samples from the US and from small children reflects a lower eukaryotic biomass and/or diversity in these samples. This hypothesis requires further investigation, but is inline with other results. Previous studies report lower bacterial diversity in western populations and in young children (reviewed in Lozupone et al., 2012). Further, lower prevalence of gut symbionts is associated with the adoption of western lifestyles (Rook, 2012), and prevalence and diversity are lower in temperate regions compared to the tropics (Bogitsh et al., 2005; Harhay et al., 2010).

### Eukaryotic microbiota in the mammalian gut

Mammals as a whole harbor a diverse community of eukaryotic microbes in their gut, and compositional differences follow host phylogeny and diet. The human gut microbioes is similar that of other mammals, particularly of primates.

Diet drives differences in bacterial community composition across mammalian species (Ley et al., 2008b; Muegge et al., 2011). We also see compositional differences according to diet in the eukaryotic communities. Herbivores make up most of our mammalian samples that successfully amplified, and are differentiated between hindgut and foregut fermenters. The presence and absence of entire lineages varies according to dietary group, for example only hindgut fermenting herbivores harbor litostome ciliates and anaerobic fungi (e.g., *Neocallimastix;* Figure 1). Lineages that are present in multiple host species such as *Blastocystis* and *Entamoeba* show species level divergence that tracks host phylogeny. Artiodactyls harbor *Entamoeba bovis*, while primates have *Entamoeba coli* and *E. hartmanii* (Table S1). Host-specificity is also observed in the distribution of *Blastocystis* subtypes (Table S1). We detected *Blastocystis* ST1, ST2, and ST3 in humans (Table 1) and also in the primates (baboon and orangutan) (Table S1). Kangaroos, foregut-fermenting herbivores, had large numbers of *Blastocystis* ST8 (Figure 1; Table S1).

**Figure 2:**
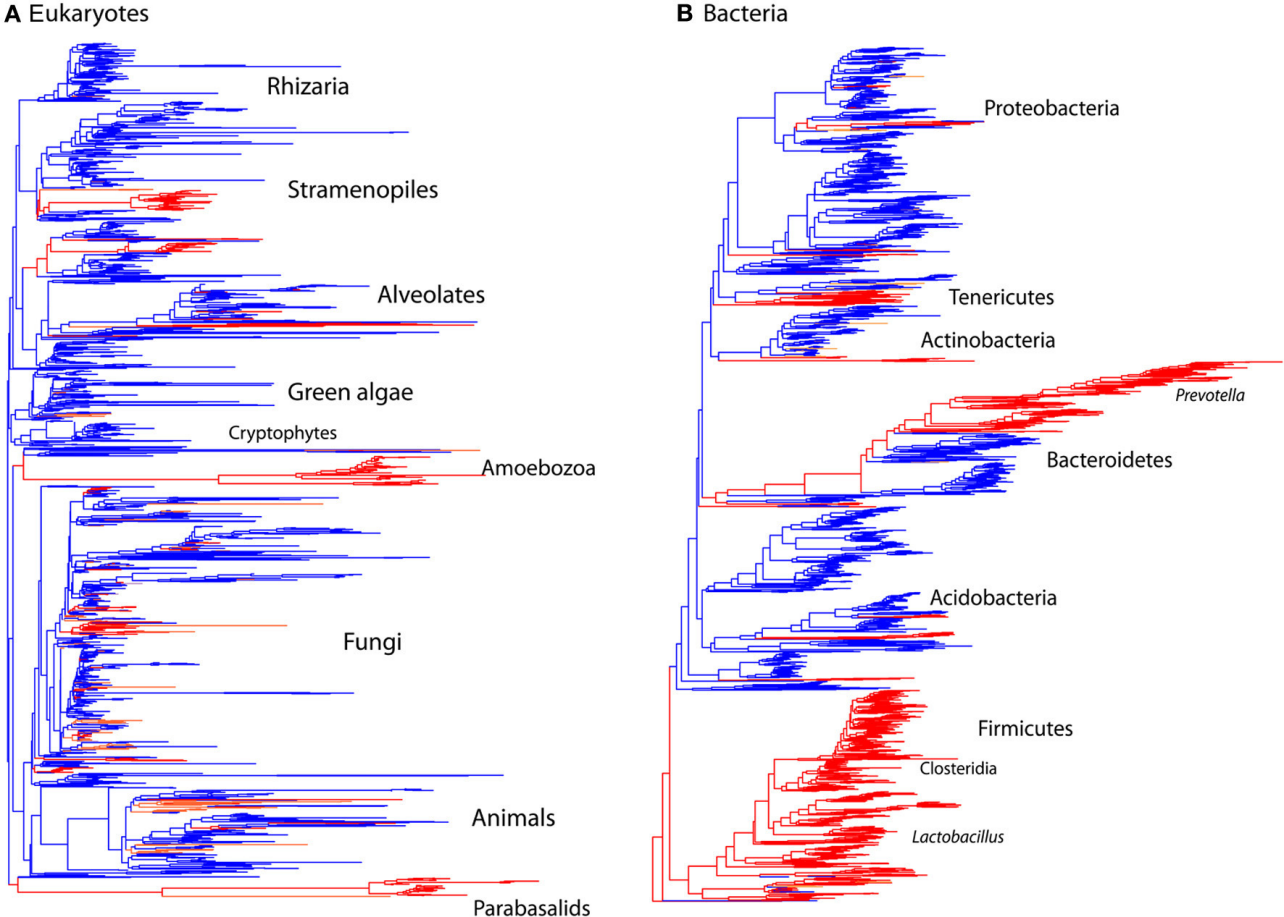
**Comparison of the phylogenetic distribution of taxa from mammalian gut to other environments**. Sequences detected in the mammalian gut come from a smaller number of lineages and have lower overall diversity compared to other environments, reflecting the limited number of lineages that have successfully colonized animal hosts. Tree contains sequences from 32 mammalian gut samples (red) and 32 samples total from skin, terrestrial, and aquatic habitats (blue). Tips present correspond to the data rarefied to 150 sequences per sample for comparison. **(A)** Eukaryotic 18S rRNA tree constructed using RAxML with the topology constrained to the SILVA 108 reference tree. **(B)** Bacterial 16S rRNA tree from Greengenes 2011 release. Branches are colored according to the environment that contributed the majority of the sequences.

Diversity patterns for eukaryotic microbes within the mammalian gut differ in two ways from those of bacteria. First, eukaryotic microbes show a patchy distribution across samples, such that the most abundant lineages in some samples are completely absent from others (Figure 1). In contrast, bacterial community composition at comparably high taxonomic levels is broadly consistent across individuals and across populations; e.g., Bacteroidetes and Firmicutes are generally the dominant phyla (Figure 1; Ley et al., 2008b; Consortium, 2012; Yatsunenko et al., 2012). Second, within a phylum-level lineage there is less diversity at the strain and species level for eukaryotes, even after controlling for differences in sequencing depth (Figure 2). This suggests that presence or absence of deep lineages may be more informative than variation at lower taxonomic levels for eukaryotes.

### Diversity of gut microbiota compared to other environments

**Figure 3:**
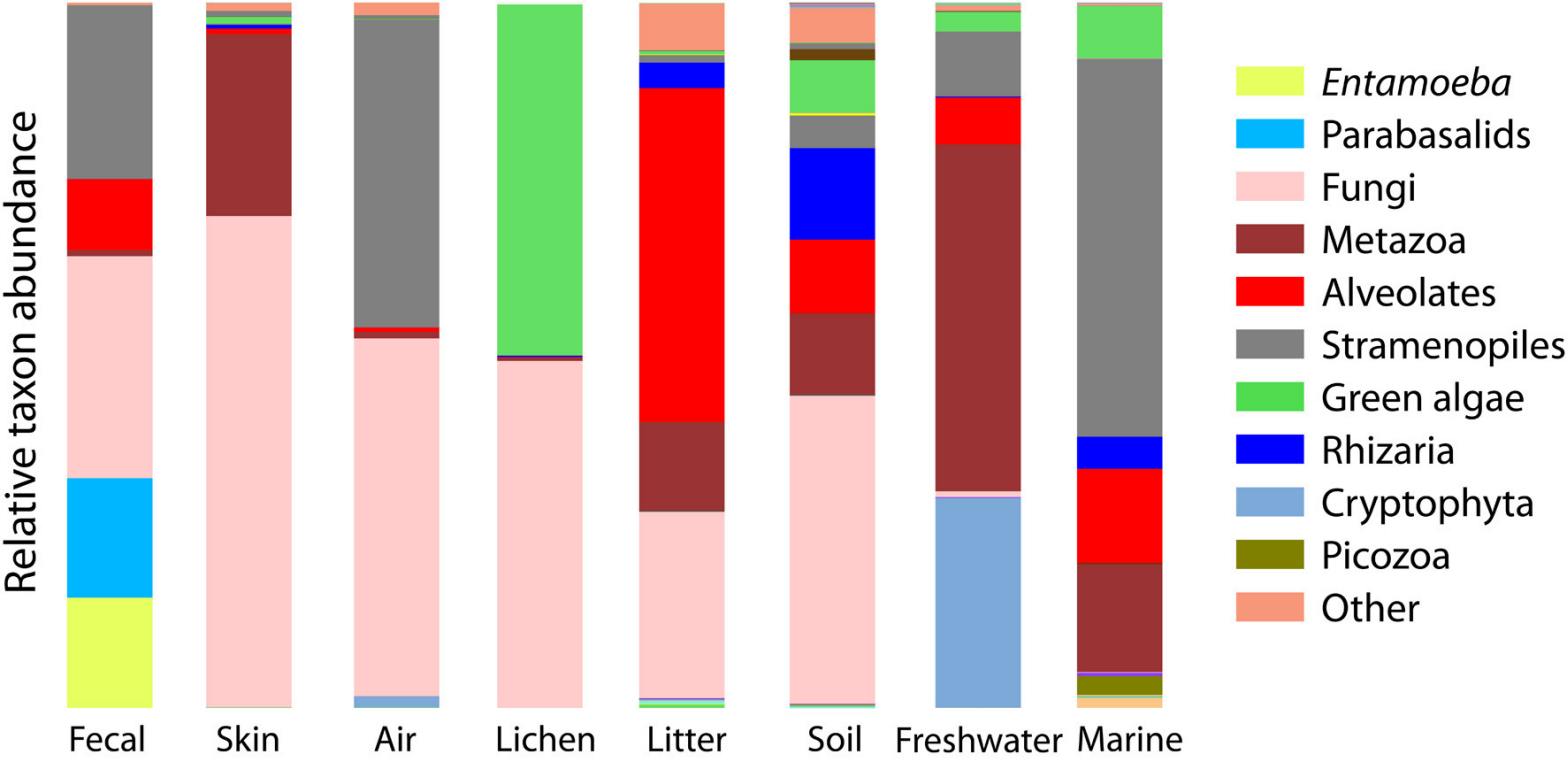
**Bar chart of the relative abundance of sequences falling into the major clades of eukaryotes depicts the overall divergence in community composition across sample types**. Major clades are the deepest divisions within eukaryotes (Parfrey et al., 2010; Adl et al., 2012) and are roughly equal to the phyla or superphyla level of bacteria.

The microbial eukaryotic communities detected in the mammalian gut are quite distinct from environmental communities both at the OTU level, as seen in the low numbers of shared OTUs (Table 2) and at higher taxonomic levels (Figures 2, 3). Just 3% of non-fungal OTUs from the gut are shared with skin, terrestrial, and aquatic environments (Table 2). The composition eukaryotic communities in the mammalian gut is significantly different than the composition found in environmental samples (ANOSIM *p* = 0.001, *R* = 0.76), and this is true for bacteria as well (ANOSIM *p* = 0.001, *R* = 0.94). Overall, beta-diversity patterns observed for eukaryotes are significant similar to bacterial beta-diversity as assessed by Mantel tests comparing the unweighted UniFrac distance matrices (*p* = 001, *R* = 0.658; *N* = 113). The distinctiveness of gut communities can also be seen when the branches of the 18S and 16S trees are colored according to the environment where the sequences were detected (Figure 2). Sequences from the gut are significantly clustered in both 16S and 18S (Figure 2) as assessed by the phylogenetic test [*p*-test *p* < 0.001; (Martin, 2002)] and UniFrac significance test (*p* < 0.001).

In accordance with previous observations, fewer lineages of eukaryotes reside in the mammalian gut than in other habitats, and those lineages that have successfully colonized the vertebrate gut have diversified as they have co-evolved with their hosts over millions of years (Parfrey et al., 2011). Similar patterns have also been observed for bacteria (Ley et al., 2006). Here, we see significantly lower levels of alpha diversity in gut communities compared to other environments for eukaryotes (*t*-test comparing Faith's phylogenetic distance in the gut vs. environmental samples: *p* < 0.001), and bacteria (*p* < 0.001).

### Eukaryotic communities associated with human skin resemble terrestrial samples

**Figure 4:**
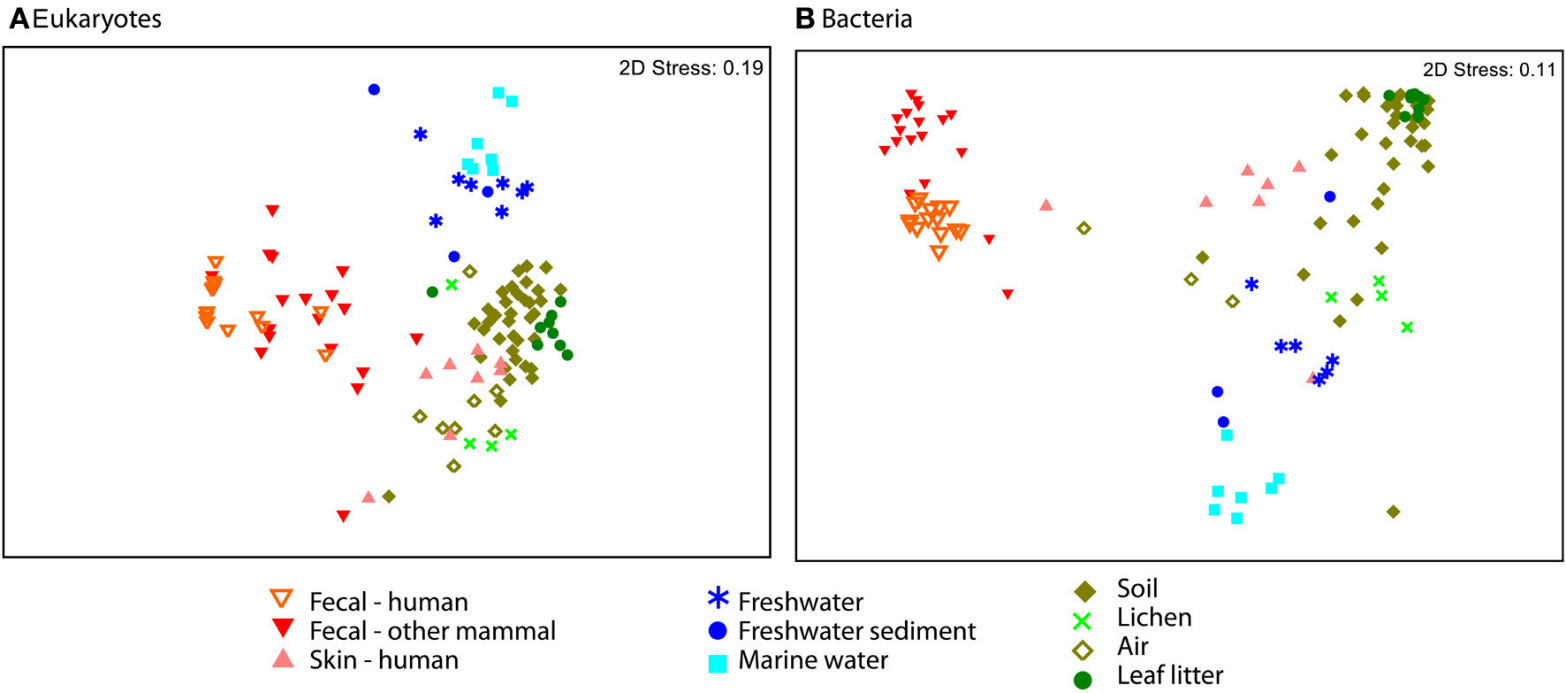
**NMDS plot of unweighted UniFrac reveal separation across major environmental categories**. Plots **(A)** Eukaryotes and **(B)** Bacteria show the distinction between fecal samples (red and orange) and those from other environments, including skin (pink). Air samples were collected over terrestrial habitats.

Eukaryotic communities associated with human skin are composed mostly of fungi and have low diversity overall, in line with expectations from other studies (Paulino et al., 2006; Findley et al., 2013). Skin samples group with terrestrial samples in NMDS plots of unweighted UniFrac (Figure 4). Similarity in the fungi detected in skin and terrestrial samples accounts for much of this similarity; 70% of the OTUs on skin are fungi, and of these more than 80% (113 OTUs) are shared with soil or other terrestrial samples. The low taxonomic resolution of fungi with the 18S marker may inflate the number shared OTUs to some extent (Schoch et al., 2012). Non-fungal OTUs detected on skin correspond to mites and a handful of low abundance OTUs that are commonly found in soil such as cercozoan flagellates. The overlap between skin and soil communities may reflect frequent contact between skin and soil, or with airborne microbes, which can have high abundances of soil-associated taxa (Bowers et al., 2011b). In support of this hypothesis, skin bacterial communities also frequently group with environmental samples (Figure 4). These results are suggestive, but are drawn from skin and soil samples taken in different locations within different studies (see Methods). Testing the hypothesis that skin communities resemble terrestrial environments because contact enables frequent dispersal requires samples from human skin and the surrounding environment, including dust and soil, collected at the same time.

### Comparison of eukaryotic communities in other habitats

Our dataset includes samples from a range of environments and enables us to compare eukaryotic communities across environmental habitats. Microbial eukaryotic communities are highly differentiated across host marine, freshwater, and terrestrial habitats as assessed by ANOSIM (Figure 4; ANOSIM *R* = 0.78, *p* = 0.001). The sample set analyzed here includes more soil and other terrestrial samples, such as lichens and leaf litter than water samples (Table S1), but the differences across habitat types persist when the data is subsampled to equal sample numbers across habitat types (see Methods). For each of the 1000 sub-sampled trials, the divide between freshwater, marine, and terrestrial environments was highly significant and explains much of the variation (ANOSIM ranges: *p* = 0.001 to 0.005 and *R* = 0.65 to 0.60). These habitats were also significantly clustered in the 18S tree (*p*-test *p* = 0.001 for each pair of environments).

Beta-diversity differences across environments are underlain by a strong differentiation in the high-level clades present across environments (Figure 3). Some clades are restricted to one type of sample, for example, Amoebozoa (*Entamoeba*) and parabasalids are characteristic of fecal samples and cryptophytes comprise a large portion of the freshwater community, while the recently identified Picozoa clade (formerly “picobiliphytes”; Seenivasan et al., 2013) is restricted to marine environments. Yet, across all environments, diversity is dominated by just a few clades. Animals, fungi, alveolates, Cercozoa, and stramenopiles make up 79% of all sequences (Figure 3). At the OTU level very few taxa are shared across habitats (Table 2).

Communities from environmental samples show a distinct separation between terrestrial and water samples, and between marine and freshwater samples in beta-diversity plots (Figure 4). In accordance with previous studies that report salinity as the most important factor structuring bacterial and archaeal community composition (Lozupone and Knight, 2007; Auguet et al., 2010; Wang et al., 2011), and we also see a major divide in bacterial community composition between freshwater vs. marine habitats (Figure 4). Eukaryotic taxa also cross the saline/non-saline boundary infrequently (e.g., Shalchian-Tabrizi et al., 2008; Logares et al., 2009; Brate et al., 2010). In our data, compositional differences between freshwater and marine eukaryotic communities are highly significant (ANOSIM *p* < 0.001, *R* = 0.58), though our dataset includes a limited number of samples. Interestingly, the difference between aquatic and terrestrial environments are also significant and explain more variation in community structure (ANOSIM *R* = 0.71 for terrestrial vs. freshwater and *R* = 0.85 for marine vs. terrestrial comparisons). Further studies that include large numbers of samples from all three habitat types, preferably from consistent geographic locations, will be necessary to determine the deepest divisions in eukaryotic community composition across environments.

## Conclusions

Our results demonstrate clearly that microbial eukaryotes are a normal component of the mammalian microbiota, and that the communities they form, although not as diverse as bacterial communities in the gut, are nonetheless diverse and correlate with key features of their hosts. Interestingly, humans with non-western diets and lifestyles are comparable to other mammals in the microbial eukaryote diversity they harbor. In contrast, humans living Western lifestyles instead have very low diversity of gut microbial eukaryotes. Whether these differences are due to diet, hygiene, level of contact with animals, host genetics, or other lifestyle factors that differ among the populations surveyed remains a topic for further work: of particular interest is whether the loss of the microbial eukaryote diversity with which we as mammals have co-evolved is a trigger for the autoimmune diseases that are far more prevalent in Western populations.

One intriguing difference between eukaryotic and bacterial communities is that eukaryotic communities in the vertebrate gut are heterogeneous across samples, whereas the dominant bacterial lineages are consistently recovered across individuals and across species. The patchy distribution of eukaryotes across individuals, combined with the host-species specificity of resident eukaryotic microbes, suggests that it will be difficult to clearly identify the healthy, or “normal,” core eukaryotic microbiota of the human gut, just as is it is also difficult to identify a core gut bacterial community shared across humans (Li et al., 2013). Consequently, future studies of microbial eukaryote communities should focus more on identifying variation that is associated with different phenotypic states, including disease states.

Finally, comparison of the mammalian gut to other environments shows that fewer deep lineages are associated with the gut than in free-living communities, and alpha diversity is lower. This pattern resembles the pattern found in bacteria in the same environments. Eukaryotes have less diversification within lineages at shallow levels than observed for bacteria, however, suggesting that although the big picture of high-level diversification is the same across these taxa, the fine-grained patterns may differ. With the improved tools for eukaryotic surveys presented here, we are now poised to characterize microbial eukaryotes across environments on a large scale in projects such as the Earth Microbiome Project, providing a much richer understanding of the relationships between pathogens, commensals, and beneficial members of our microbial eukaryote community.

### Conflict of interest statement

Several authors on this manuscript are, or were at the time of the study, employees of 454 Life Sciences, a Roche Company (Branford, CT), whose technology is used in this study.

## Supplementary material

Figure S1 **Rarefaction curves with alpha diversity metric PD Whole tree**. Rarefaction curves are approaching an asymptote indicating diversity has been adequately captured, especially for fecal samples. Error bars are standard deviation. **(A)** Eukaryotes and **(B)** Bacteria.
