# Supplementary figures and images for: Communities of microbial eukaryotes in the mammalian gut within the context of environmental eukaryotic diversity
Source: Front Microbiol. 2014 Jun 19;5:298. doi: 10.3389/fmicb.2014.00298 (PMC4063188; doi:10.3389/fmicb.2014.00298)

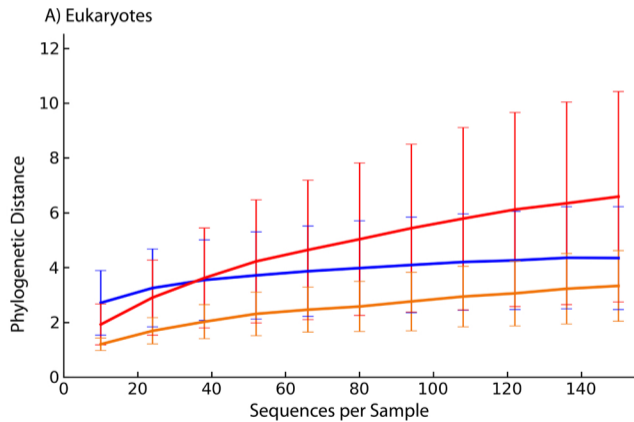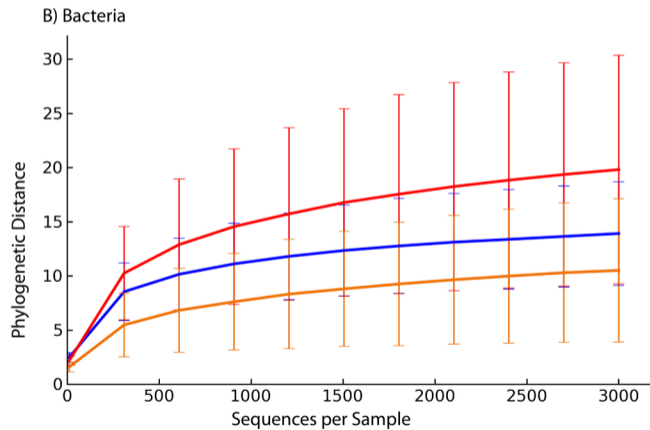

Environmental

Fecal

Skin

Supplement: Figure S1 — Rarefaction curves with alpha diversity metric PD Whole tree. Rarefaction curves are approaching an asymptote indicating diversity has been adequately captured, especially for fecal samples. Error bars are standard deviation. (A) Eukaryotes and (B) Bacteria. [file Presentation1.PDF]
